# Ionically Paired Layer-by-Layer Hydrogels: Water and Polyelectrolyte Uptake Controlled by Deposition Time

**DOI:** 10.3390/gels4010007

**Published:** 2018-01-11

**Authors:** Victor Selin, John F. Ankner, Svetlana A. Sukhishvili

**Affiliations:** 1 Department of Materials Science & Engineering, Texas A&M University, College Station, TX 77843, USA; vselin@tamu.edu; 2 Spallation Neutron Source, Oak Ridge National Laboratory, Oak Ridge, TN 37831, USA; anknerjf@ornl.gov

**Keywords:** polyelectrolytes, layer-by-layer, multilayers, neutron reflectometry, exponential growth

## Abstract

Despite intense recent interest in weakly bound nonlinear (“exponential”) multilayers, the underlying structure-property relationships of these films are still poorly understood. This study explores the effect of time used for deposition of individual layers of nonlinearly growing layer-by-layer (LbL) films composed of poly(methacrylic acid) (PMAA) and quaternized poly-2-(dimethylamino)ethyl methacrylate (QPC) on film internal structure, swelling, and stability in salt solution, as well as the rate of penetration of invading polyelectrolyte chains. Thicknesses of dry and swollen films were measured by spectroscopic ellipsometry, film internal structure—by neutron reflectometry (NR), and degree of PMAA ionization—by Fourier-transform infrared spectroscopy (FTIR). The results suggest that longer deposition times resulted in thicker films with higher degrees of swelling (up to swelling ratio as high as 4 compared to dry film thickness) and stronger film intermixing. The stronger intermixed films were more swollen in water, exhibited lower stability in salt solutions, and supported a faster penetration rate of invading polyelectrolyte chains. These results can be useful in designing polyelectrolyte nanoassemblies for biomedical applications, such as drug delivery coatings for medical implants or tissue engineering matrices.

## 1. Introduction

Polyelectrolyte multilayers (PEMs) are traditionally made by sequential deposition of oppositely charged polymers at a surface using the layer-by-layer (LbL) technique [1]. In addition to ionically pairs, cooperative interactions that lie at the heart of many LbL systems—secondary interactions such as hydrogen bonding, van der Waals forces, and hydrophobic interactions—can also play important role in LbL assembly [2,3,4]. The cooperative type of intermolecular interactions is quite universal and can be applied to metal-protein [5] and hydrogen bonding systems [6]. The applicability of the LbL technique to a wide range of polymers and substrates, and the ease of incorporation of highly functional molecules within these films, have excited interest in a wide range of applications including tissue engineering [7], in which LbL assemblies can be used to enhance tissue regeneration [8,9]. Moreover, PEMs show particular promise for surface functionalization of biomedical devices to create hemocompatible, antibacterial, and antioxidant surfaces [10], or to control cell adhesion-localized delivery of bioactive molecules [11,12,13]. Future proposed applications of PEMs as bioactive matrices or drug molecule carriers dictate certain requirements for film capacity and functionality, defined by the overall film thickness and controllable swelling. In particular, it becomes increasingly desirable to achieve a film thickness on the order of microns upon the deposition of a small number of bilayers. In addition, well-controlled interaction with water, i.e., swelling, can enable controlling the dosage of an active component released from the film. 

Depending on the binding strength between the components [13,14], and therefore the mobility of polymer chains during LbL film deposition, two main types of film growth can be distinguished. Namely, linear LbL growth describes those systems that exhibit a constant increment in thickness with deposition of successive bilayers and is usually a characteristic of strongly paired polyelectrolytes and/or nanoparticles [15,16]. On the other hand, nonlinear films demonstrate an increase in deposited film mass per deposition step, reflecting the high mobility of chains during deposition and the resulting penetration of the invading component into the film [17,18,19]. This growth mode has been predominantly reported for more weakly bound systems, and can be additionally controlled by several parameters. One of these parameters is polymer molecular weight—a parameter that is directly related to mobility of polymer chains within the films [20,21]. For ionically paired PEMs, the mobility of chains also can be regulated by the introduction of small ions [22,23]. Moreover, in the case of weak polyelectrolytes, ionization of polymer chains, determined by solution pH and assembly conditions, can also affect chain mobility and film growth [24,25].

The literature fundamentally agrees that the main factor causing exponential growth is enhanced diffusivity of polymer chains [26,27,28]. For more strongly bound films, the invading component is kinetically frozen at the outermost PEM layer and does not diffuse deeply, resulting in linear film growth with a small incremental mass increase that corresponds to monolayer coverage. The original mechanism of exponential growth was attributed to an “in-and-out” diffusion of polymer chains throughout the entire film thickness [28]. This mechanism was later modified to include chain diffusivity in systems that do not exhibit diffusion throughout the whole film [15,26,29,30]. Very recently, another mechanism of exponential growth has been proposed by Schlenoff that describes nonlinear deposition as a consequence of the diffusion of ionic binding sites rather than mass diffusion of the polymers [31].

Our group recently conducted a study of nonlinear growth where the deposition time used for adsorption of each individual layer was not fixed, but instead polymer adsorption and absorption was followed up to complete saturation of the film with the incoming polyelectrolyte [32]. At saturation, nonlinear films exhibited high (up to 4-fold of dry thickness) swelling ratios and were reminiscent of substrate-bound ionically crosslinked gels. We have demonstrated that as more time was allowed for an incoming polyelectrolyte to penetrate the film, film swelling continuously increased, and more time was required to reach saturation for larger layer numbers. In this work, we studied a more practical scenario, in which deposition times were fixed for all layers within the film, but varied between different films. While previously, by applying the neutron reflectometry (NR) technique [33,34,35,36,37,38] to dry LbL films, we demonstrated that layer deposition time controlled film internal structure [32]; here, we study swelling of LbL films constructed using fixed deposition time and explore how the internal film structure affects kinetics of polyelectrolyte chain invasion. Moreover, we explore the effect of layer deposition time on film stability in salt solutions. This study further contributes to the understanding of the relationship between growth conditions of non-linear PEMs and the behavior of these films in aqueous solutions containing small ions and/or polyelectrolytes. 

## 2. Results and Discussion

### 2.1. Film Swelling, Stability in Salt, and Poly(Methacrylic Acid) (PMAA) Ionization as a Function of Layer Deposition Time

**Figure 1:**
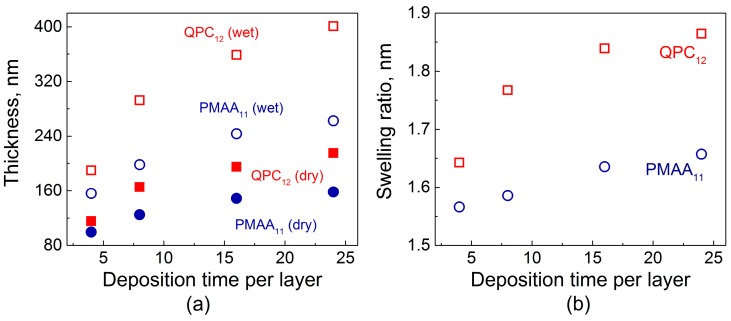
(**a**) Dry and in situ thicknesses of PMAA_11_ (poly(methacrylic acid),red symbols) and QPC_12_ (quaternized poly-2-(dimethylamino)ethyl methacrylate, blue symbols) films as a function of deposition time per layer; (**b**) Calculated swelling ratio of PMAA_11_ (red symbols) and QPC_12_ (blue symbols) films as a function of deposition time per layer.

Figure 1 shows the total dry film thickness as a function of deposition time per layer. To compare the increment in thickness during film growth for each deposition time, two samples were deposited simultaneously, terminated with either poly(methacrylic acid) (PMAA) or quaternized poly-2-(dimethylamino)ethyl methacrylate (QPC). Figure 1a illustrates that the total amount of material absorbed increases with deposition time per layer. The dry thickness of both PMAA_11_^24min^ and QPC_12_^24min^ films was more than 1.5× greater than that for the films made using an 8 min deposition time. Similar observations by Schlenoff and co-workers were made for a different polyelectrolyte system studied at high salt concentrations: when the polyelectrolyte deposition time increased from 3 to 20 min, a 1.5-fold increase in film thickness was observed [23]. The thicknesses of QPC-terminated films were greater in all cases, with a tendency to increase with time. Moreover, comparison of the thickness ratios QPC_12_^4min^/PMAA_11_^4min^ and QPC_12_^24min^/PMAA_11_^24min^ gives 1.1 and 1.35, respectively, reflecting a strong dependence of mass absorbed on exposure time and therefore confirming the diffusive nature of growth in non-linear PEMs, also described in our previous work [32].

Figure 1b shows that not only the amount of polymers uptaken by the film at each deposition step but also the degree of film swelling was strongly affected by the deposition time per layer. The swelling ratio was determined as the ratio of wet film thickness, measured by in situ ellipsometry in the presence of buffer above the growing film, to the thickness of the dry films. Longer layer deposition times allowed for greater film intermixing, charge overcompensation, and enhanced creation of QPC loops, which are formed by QPC units that do not participate in the formation of ionic pairs with PMAA and carry a permanent charge [32]. The osmotic pressure created by the counterions, electrostatic and steric repulsion between excess charge in the loops, as well as the film hydration all contribute to an uptake of large amounts of water into the films. In addition, film swelling was higher for QPC-capped films. On the contrary, the penetration during deposition of PMAA, which is a weak polyelectrolyte, resulted in lower swelling, as a charge in the PMAA loops was suppressed by the greater amount of the polyacid accommodated within the films [32,39]. 

**Figure 2:**
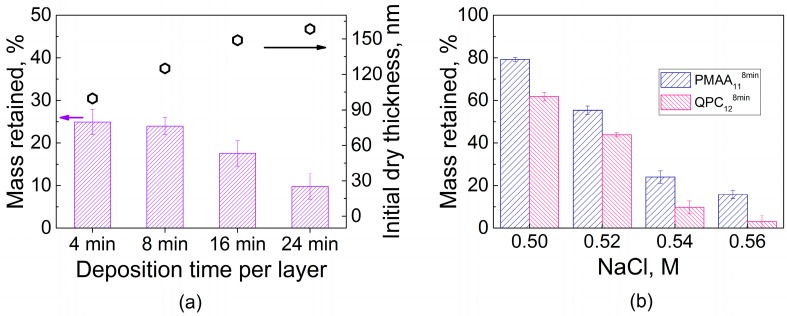
(**a**) Total film thicknesses and stability of PMAA_11_ films assembled using different deposition times per layer upon post assembly overnight exposure to 0.54 M NaCl; (**b**) stability of PMAA_11_^8min^ and QPC_12_^8min^ films within NaCl solutions of various concentrations after overnight exposure.

The amount of water penetrating into the films intuitively should be related to binding strength between assembled polyelectrolytes. One way to characterize the polyelectrolyte binding strength is to compare the stability of LbL films in different salt solutions, where small ions compete with electrostatic pairs formed by polymer association. Exposure of PEMs to salt concentrations lower than those of film dissolution promotes polymer diffusion and causes film smoothening as shown and quantified by atomic force microscopy for systems formed by strong and weak polyelectrolytes [40,41]. Notably, prior work on PEM films of strong polyelectrolytes, often composed of strongly electrostatically pairing polystyrene sulfonate (PSS), has shown that these films can sustain relatively high salt concentrations, up to 3.5 M NaCl [40,42]. Such concentrations of salt can disassemble PEM films if they are formed by weak polyelectrolytes, such as PAA [43] or the PMAA used in this work. To avoid film dissolution, much lower salt concentrations (0.1–0.4 M) were used to study enhanced polyelectrolyte chain dynamics within QPC/PMAA films [35]. Figure 2 shows that higher salt concentrations (such as a 0.54 M NaCl solution) lead to disassembly of QPC/PMAA films. The dry thickness of LbL films assembled using various deposition times per layer was measured after overnight exposure to a 0.54 M NaCl solution. The decrease in thickness results from binding site disruption and subsequent loss of material. The rate of desorption of polyelectrolytes from the film was not affected by the film deposition time, thickness, and/or internal structure and was approximately the same (~3–4 nm/min, data not shown) for all films. However, the equilibrated mass loss was larger for thicker PMAA_11_^24min^ films when compared to thinner PMAA_11_^4min^ films (~90% and ~75% of the original thickness, respectively) after exposure to 0.54 M NaCl for 24 h (Figure 2a). This effect demonstrates that increased deposition time per layer results in a decrease in film stability in salt solutions. Shorter deposition times produce better-layered structures, with fewer polymer units included in loops and a larger number of ionic pairs per single polymer chain, which can withstand salt ion assaults better. In contrast, longer deposition times produce highly intermixed nonlinear PEMs that are more prone to disassembly in salt solutions. Figure 2b compares mass retained after an overnight immersion of PMAA-capped and QPC-capped films in solutions with different salt concentrations. Both films showed the same expected trend of decreasing stability with increasing salt concentration, but films capped with QPC in all salt concentrations were less stable than PMAA-capped assemblies. This observation shows that it might be possible to control film stability via QPC immersion. 

**Figure 3:**
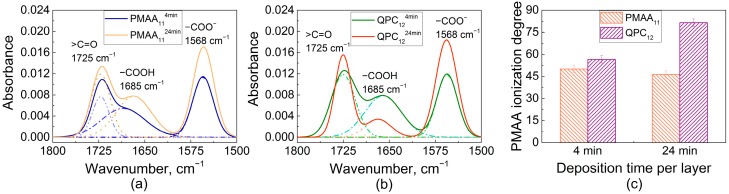
Fourier-transform infrared spectroscopy (FTIR) spectra of dry PMAA_11_^4min^ and PMAA_11_^24min^ (**a**); FTIR spectra of dry QPC_12_^4min^ and QPC_12_^24min^ (**b**); calculated values of PMAA ionization degree within QPC_12_^4min^, QPC_12_^24min^, PMAA_11_^4min^ and PMAA_11_^24min^ films (**c**).

An explanation for this effect was then sought in the higher swelling of QPC-capped films (Figure 1b), and ionization of assembled PMAA was determined using FTIR. Earlier, FTIR was used by several groups, including ours, to study the ionization of weak polyelectrolytes assembled within linear PEMs [32,39,44,45]. Figure 3a shows FTIR spectra of PMAA_11_^4min^ and PMAA_11_^24min^ films. Both spectra show vibrational bands at 1725 cm^−1^ and 1568 cm^−1^ associated with the stretching vibrations of carbonyl vibrations of QPC (ν, >C=O) and asymmetric stretching vibrations of the carboxylate groups (ν_as_, −COO^−^), respectively, as well as a peak at 1685 cm^−1^ associated with the vibrations of protonated carboxylic groups (−COOH). The ionization degree of PMAA within the assembled multilayers was calculated as the ratio of the area of −COO^−^ to the sum of −COOH and −COO^−^ absorbances, assuming equal extinction coefficients for vibrations associated with these bands [46]. The ionization degrees of PMAA were 50 ± 2% and 46 ± 2% for PMAA_11_^4min^ and PMAA_11_^24min^ films, respectively. A layer of QPC was then deposited on these films and the resulting QPC_12_^4min^ and QPC_12_^24min^ films were measured again (Figure 3b), yielding PMAA ionization of 57 ± 2% and 82 ± 2%, respectively. QPC taken up by the film causes an increase in the average ionization of assembled PMAA chains due to the formation of ion pairs between QPC and previously uncharged PMAA units, while the addition of PMAA suppresses the ionization of PMAA in the adsorbed polymer loops, likely because of the accumulation of an excess of negative polymer charge within the film. Previously, it was shown by several groups that complexation of weak polyelectrolytes in PEMs lowers the pKa and alters the ionization of carboxylic groups [32,44,47,48]. The effect of capping layer on ionization of assembled weak polyelectrolytes was previously reported for PEMs that demonstrate linear growth [49,50]. Here, we show that the capping layer also impacts ionization of weak polyelectrolytes in non-linear LbL films. 

It is also clearly seen that ionization of PMAA is strongly dependent on deposition time (Figure 3c), and larger amounts of QPC taken up lead to higher charge in assembled PMAA chains. As shown in Figure 2, however, larger deposition time per layer decreases the stability of LbL films in salt solutions, suggesting that ionization of PMAA within the films is inversely correlated to films stability. We suggest that a key to lower salt stability of the films constructing using longer deposition times is their larger swelling, caused by more loopy conformations of QPC. In highly swollen films, loopy QPC chains have fewer binding points with PMAA and accumulate excess charge, increasing osmotic pressure within the film. As a result, the net interaction energy between polyelectrolytes decreases, leading to faster chain dynamics.

### 2.2. Effect of Film Internal Structure on Polyelectrolyte Uptake: Neutron Reflectometry Studies

**Figure 4:**
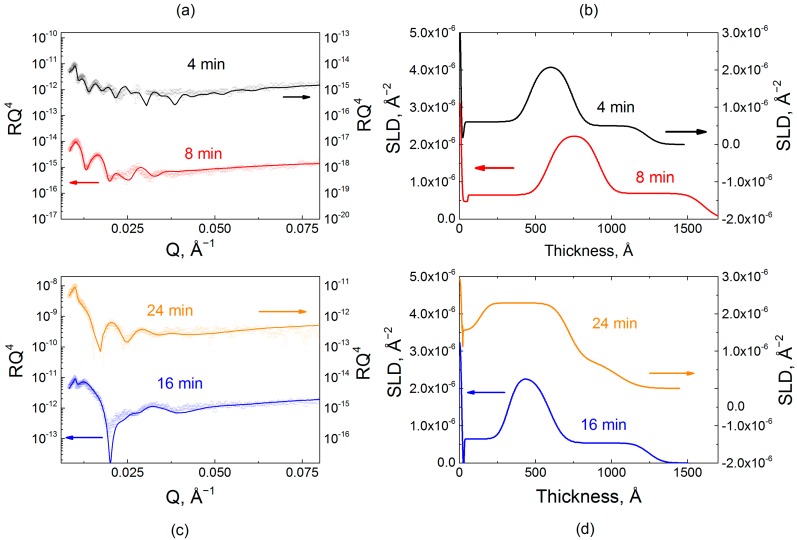
Reflectometry data (**a**,**c**) and scattering length density profiles for PMAA/QPC films formed using hydrogenated and deuterated components; (**b**) *SLD* profiles of films assembled using *d*PMAA as a marker layer with layer sequences QPC_8_/*d*PMAA_9_/QPC_14_^4min^ and QPC_8_/*d*PMAA_9_/QPC_14_^8min^, (black and red lines, respectively); (**d**) *SLD* profiles of films assembled using *d*PMAA as a marker layer with layer sequences QPC_4_/*d*PMAA_5_/QPC_10_^16min^ and QPC_4_/*d*PMAA_5_/QPC_8_^24min^ for samples with 16 and 24 min deposition times (blue and orange lines, respectively).

**Figure 5:**
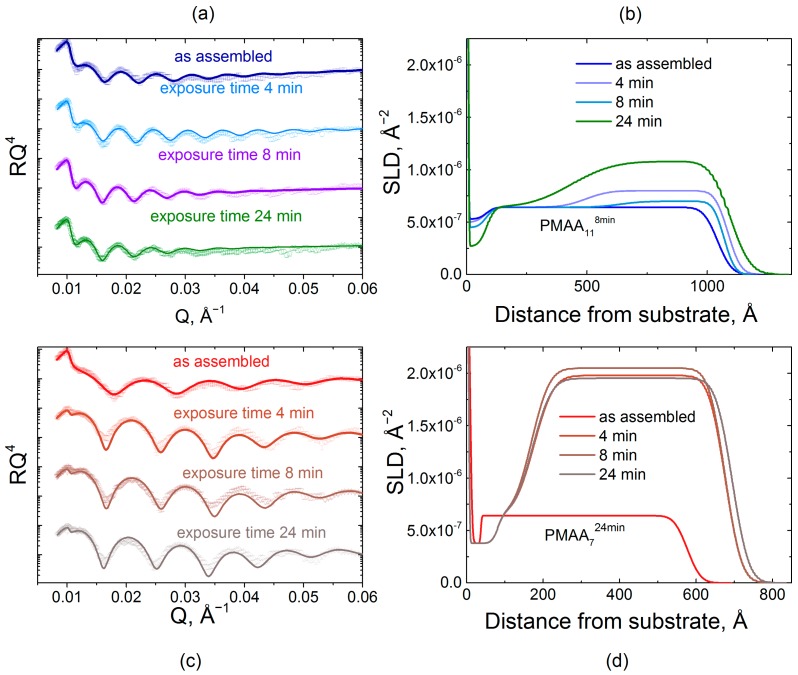
The effect on the film internal structure of the exposure of hydrogenated PMAA_11_^8min^ and PMAA_7_^24min^ films to a 0.2 mg/mL *d*QPC solution, as illustrated by neutron reflectometry. Reflectometry data (plotted as RQ^4^ to enhance small features) (**a**,**c**) and scattering length density profiles (**b**,**d**) for PMAA_11_^8min^ and PMAA_7_^24min^ films exposed to *d*QPC solution for 4, 8, and 24 min in phosphate buffer at pH 6.0.

**Figure 6:**
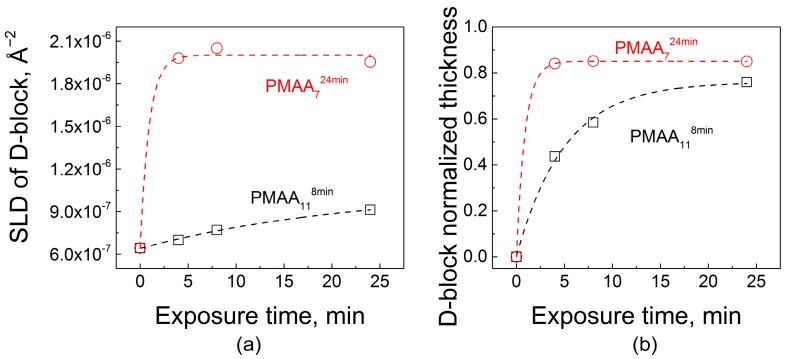
A comparison of the effect on film internal structure of the exposure of hydrogenated PMAA_11_^8min^ and PMAA_7_^24min^ films to a 0.2 mg/mL *d*QPC solution, as illustrated by fitted scattering length density (**a**) and the fitted thicknesses of deuterated block (**b**).

A central point of this work is an exploration of how assembly conditions of non-linear LbL films, and therefore internal film structure, affect invasion of polyelectrolyte chains. To that end, we assembled films with different internal structures, using deposition times that were fixed for all layers within the same film, and varied between various films. In the first set of experiments, which sought to quantify the internal structure of the films using NR, a layer of *d*PMAA was incorporated within the film for contrast (Figure 4). After establishing the required film thicknesses, a number of layers, and quantifying layer intermixing, *d*PMAA was substituted with hydrogenated PMAA for film construction, and fully hydrogenated films, assembled with different deposition time per layer, were exposed to a solution of *d*PMAA to study the effect of internal film layering on the uptake of invading chains. The results of the latter ‘chain invasion’ experiments are shown in Figure 5 and Figure 6. In both cases, the sample design was dictated by the requirements of the NR technique. Thus, knowing that the thickness of deposited films is a function of deposition time, the number of bilayers was altered for 4, 8, 16, and 24 min films. Because the amount of material deposited depended strongly on immersion time and, due to instrumental resolution, the total film thickness in NR should not exceed ~350 nm, films constructed using longer deposition times by design had fewer polyelectrolyte layers. Upstream collimation determines the angular divergence of the incident beam (*δθ*) which, in turn, is the dominant term in the instrumental resolution of the SNS-LR (*δQ/Q* = *δθ/θ*, where *θ* is the angle of incidence onto the sample). The maximum resolvable film thickness is given by *d*_max_ = 2*π/δQ*, which is about 350 nm for these measurements.

Figure 4 shows reflectivity data and fitted profiles for a series of samples containing incorporated *d*PMAA marker layers (i.e., QPC_8_/*d*PMAA_9_/QPC_14_^4min^, QPC_8_/*d*PMAA_9_/QPC_14_^8min^, QPC_4_/*d*PMAA_5_/QPC_10_^16min^ and QPC_4_/*d*PMAA_5_/QPC_8_^24min^ films) with designed varied internal structure controlled by layer deposition time. The polymer layers included in the reflectivity model consisted of the BPEI priming layer and three hydrogenated/deuterated/hydrogenated stacks. The SLD of the hydrogenated stacks was constrained to be the value for the hydrogenated matrix determined in an independent measurement. The SLD of the deuterated block was found by fitting the reflectivity data (Tables S1–S4). Figure 4 highlights the dramatic effect of layer deposition time on internal film structure. As seen from the SLD profiles, the deuterated marker layers appear as peaks in SLD, whose shape indicates the amount of material deposited, its position, and its spatial extent within the layer. Importantly, the value of the SLD of the deuterated block has almost the same value, 2.33 × 10^−6^ Å^−2^, for all four samples and is independent of the deposition time per layer and sample design. 

Based on *d*PMAA chain diffusivity, allowing more time per layer deposition encouraged penetration of PMAA chains into neighboring layers. This effect is revealed by a change in interfacial width and deuterated block thickness as a function of deposition time. Thus, based on chain diffusivity, 4 and 8 min deposition times do not allow chains to penetrate much into the hydrogenated bulk. However, increasing deposition time to 16 and 24 min causes a noticeable change in chain intermixing. Tracking the increase in deposited polymer mass, the interfacial full width, *σ*_int_, between hydrogenated and deuterated blocks increased from 6.7 to 16.7 nm for *d*PMAA, indicating a greater intermixing between hydrogenated and deuterated stacks. Continuing this trend, 24 min immersion allows enough time for *d*PMAA to penetrate completely through the film, down to a dense layer (entangled with BPEI) near the substrate, as described in our previous work [32]. 

Figure 5 illustrates penetration of deuterated *d*QPC chains into the hydrogenated matrix constructed using different deposition times per layer. Specifically, hydrogenated matrices were assembled using 8 and 24 min immersions per layer (PMAA_11_^8min^ and PMAA_7_^24min^). These films were immersed in a 0.2 mg/mL solution of *d*QPC, then taken out of solution, rinsed with buffer, and dried prior to the NR measurement. The model used to fit the NR data initially consisted of only two stacks—a priming layer and a hydrogenated matrix. Upon absorption of deuterated chains, an additional stack representing the hydrogenated matrix enriched with deuterated polymers was introduced into the model. Figure 5 shows NR data and the calculated *SLD* profiles of PEMs constructed using per-layer deposition times that were constant for each individual film but varied between different PEMs, specifically 8 and 24 min (Tables S5–S12). For both deposition times (PMAA_11_^8min^ and PMAA_7_^24min^), upon exposure to the deuterated polymer solution, the reflectivity oscillation minima shifted to lower *Q* values indicating an increase in total film thickness. The fitted NR profiles are shown in Figure 5b,d.

Polycation penetration into the two films was strikingly different. The addition of *d*QPC to the films is observed by the increase in *SLD* values, which, at shorter times, occurred only within the near-surface region of the PMAA_11_^8min^ film. The more tightly bound and structured PMAA_11_^8min^ matrix demonstrated only a gradual increase of *SLD* for the second block at short exposure times. As exposure time increased, an enhanced *SLD* zone propagated deeper into the film until the deposited polymer was distributed throughout the film. A thin layer impermeable to *d*QPC was present at the substrate in both systems. The presence of a ~8 nm-thick impermeable zone was previously observed and rationalized as a single bilayer that is strongly bound to the substrate and could not be replaced by deuterated chains [32]. Figure 6a shows a quantitative analysis of NR data upon *d*QPC penetration. The initial values of *SLD* correspond to zero time of exposure and are equal to the *SLD* of the hydrogenated block. Quantitatively, after 4 min of exposure to *d*QPC solution, the *SLD* of the block enriched with deuterated material increased by 8% and 68% for PMAA_11_^8min^ and PMAA_7_^24min^ films, respectively. Adsorption of the deuterated polycation was also accompanied by an increase in the overall film thicknesses, as seen in the NR profiles. Relatively slow dynamics of penetration of polymer chains within PMAA_11_^8min^ films can be explained by the higher degree of layering and higher density of barriers in the *d*QPC penetration path. In drastic contrast, penetration into the PMAA_7_^24min^ matrix was enhanced in comparison with penetration into the PMAA_11_^8min^ films. As seen in Figure 6, even for the shortest *d*QPC exposure time of 4 min, the polymer permeated the entire film, and a waiting time of 24 min was sufficient for the film to become saturated with the polycation. Note that the time scale of polycation invasion within PMAA_7_^24min^ film was comparable to that of penetration of QPC into nonlinear films assembled using times required to reach saturation at each deposition step [32]. Both the high degrees of swelling hydrogenated films assembled with longer deposition times (Figure 1b), as well as differences in film layering (Figure 4), might have contributed to faster penetration kinetics of *d*QPC within PMAA_7_^24min^ films. The difference in the degree of swelling of PMAA_11_^8min^ and PMAA_7_^24min^ films is not large (swelling rations 1.6 and 1.7, respectively), however. It is likely that the strikingly different internal structure and layering of PMAA_11_^8min^ and PMAA_7_^24min^ films seen in Figure 4 determined the large differences in the kinetics of uptake of polyelectrolyte chains within LbL films (Figure 6).

## 3. Conclusions

In this study, we have examined and quantified the effect of deposition time on growth, salt stability, and the invasion of polyelectrolyte chains within nonlinear LbL films. The structure of the electrostatically assembled nonlinear films exhibited a strong dependence on the length of time they were exposed to polyelectrolyte solutions during each deposition step. The swelling of the multilayers as measured by spectroscopic ellipsometry correlated well with the degree of chain intermixing within the films. Increased film deposition time resulted in highly swollen films that resemble physically crosslinked hydrogels bound to a solid substrate. Importantly, we found that the degree of layering within the films affected the stability of these films in salt solutions and pre-determined the uptake rate of newly added polyelectrolyte chains. More intermixed films assembled using longer deposition times were more swollen in water, exhibited lower stability in salt solutions, and supported faster penetration of invading polyelectrolytes. Taken together, these results demonstrate that the deposition time used during the assembly of the nonlinearly grown LbL films is a flexible and an easy way to control not only the film structure and swelling, but also to program how films interact with small ions or polyelectrolytes.

## 4. Materials and Methods 

Branched polyethyleneimine (BPEI) with *M*_w_ = 25 kDa and *M*_w_/*M*_n_ = 2.50 was purchased from Sigma-Aldrich (St. Louis, MO, USA). Hydrogenated polymethacrylic acid (*h*PMAA, or PMAA) with *M*_w_ 180 kDa and *M*_w_/*M*_n_ *=* 1.02 was purchased from Polymer Standard Services (PSS) GmbH (Mainz, Germany). Deuterated poly(2-(dimethylamino)ethyl methacrylate) (*d*PDMAEMA, *d*_15_) with *M*_w_ 90 kDa and *M*_w_/*M*_n_ 1.8, as well as deuterated PMAA (*d*PMAA) with molecular weight 180 kDa and *M*_w_/*M*_n_ < 1.1, were purchased from Polymer Source, Inc. (Dorval, QC, Canada). Hydrogenated 2-(dimethylamino)ethyl methacrylate monomer (DMAEMA), ethyl 2-bromoisobutyrate (EBiB), CuBr and 1,1,4,7,10,10-hexamethyltriethylenetetramine (HMTETA), hydrogenated and fully deuterated methyl sulfate (*d*_6_), as well as all solvents, were purchased from Sigma-Aldrich (St. Louis, MO, USA). Ultrapure Milli-Q water (Merck Millipore, Burlington, MA, USA) with a resistivity of 18.2 MΩ/cm was used in all experiments. All other chemicals were purchased from Aldrich and used without further purification.

### 4.1. Polycation Synthesis and Characterization

Hydrogenated poly(2-(dimethylamino)ethyl methacrylate) (*h*PDMAEMA) homopolymer was synthesized by atom transfer radical polymerization (ATRP) as previously described [51]. In brief, DMAEMA (1.86 g), EBiB, CuBr, and HMTETA were mixed in 8 mL of 2-propanol, at a molar ratio of 150:1:1:2, respectively. The solution was stirred continuously in an argon atmosphere at room temperature for 12 h. The polymerization was terminated with liquid nitrogen and the solution diluted with THF. The copper salts were purified by passage through a basic aluminum oxide column. The polymer was precipitated in cold hexane and then dried in a vacuum oven at 25 °C overnight. Gel permeation chromatography (GPC) analysis of *h*PDMAEMA was performed in DMF with polystyrene (PS) standards. The *M*_w_ and *M*_w_*/M*_n_ of the homopolymer were 90 kDa and 1.10, respectively, as determined by GPC. Quaternization of *h*PDMAEMA to obtain a 100% quaternized polycation with a molecular weight of 95 kDa, abbreviated here as *h*QPC, was carried out at room temperature. To synthesize *h*QPC, *h*PDMAEMA was dissolved in a mixture of ethanol/benzene (*v*:*v* = 3:1), and a stoichiometric amount of hydrogenated dimethyl sulfate was added to the solution. The mixture was stirred at room temperature overnight (Figure S1). The precipitated product was washed with acetone three times and dried under vacuum overnight. A similar procedure was carried out to synthesize deuterated quaternized polycation (*d*QPC). To that end, *d*PDMAEMA was treated with fully deuterated rather than hydrogenated methyl sulfate. The degree of quaternization was determined by ^1^H-NMR in D_2_O at pH 9 as described elsewhere [34]. Briefly, after quaternization with dimethyl sulfate, a new peak at 3.3–3.4 ppm appeared, reflecting successful quaternization of the dimethylamino proton with a methyl group. The absence of a peak C at *δ* 2.3–2.5 ppm and a peak at *δ* 4.2–4.4 ppm, which both correspond to dimethylamino protons in *h*PDMAEMA, indicates complete quaternization of *h*PDMAEMA homopolymer (Figure S2).

### 4.2. Multilayer Buildup and Polyelectrolyte Uptake Experiments

LbL films were deposited on silicon wafer substrates (111 orientation, Institute of Electronic Materials Technology, Warsaw, Poland). Prior to film deposition, silicon wafers were cleaned as described elsewhere [23] and primed with a monolayer of BPEI adsorbed from 0.2 mg/mL solution at pH 9 for 15 min. PEM film was then deposited by sequential dipping in 0.2 mg/mL PMAA and QPC solutions in 0.01 M phosphate buffer at pH 6.0 for 4, 8, 16, or 24 min. In between polymer deposition steps, the wafers were rinsed by immersing twice in 0.01 M phosphate buffer solutions at pH 6.0 for 2 min. The procedure was repeated until the required number of layers was reached. Deposition time per cycle during PEM assembly is denoted as a superscript with the number of minutes used for deposition written next to the layer name (Scheme S1). The number of layers in the PEM films is denoted by a subscript. Thus, PMAA_7_^16min^ denotes a PEM film assembled using 16 min per step and terminated at layer number 7, which is PMAA. 

For the polyelectrolyte uptake studies using neutron reflectometry (NR), hydrogenated films PMAA_11_^8min^ and PMAA_7_^24min^ were exposed to 0.2 mg/L solutions of *d*QPC in 0.01 M phosphate buffer at pH 6.0 for different time intervals. After rinsing with 18.2 MΩ/cm Milli-Q water and drying under nitrogen flow for 5 min, NR measurements were performed, and then the films were returned to the polymer solution for continued polymer uptake. Samples for internal structure studies were assembled using 4 and 8 min deposition times per immersion cycle having the design QPC_8_/*d*PMAA_9_/QPC_14_ or 16 and 24 min deposition times with design QPC_4_/*d*PMAA_5_/QPC_10_ and QPC_4_/*d*PMAA_5_/QPC_8_, respectively. The difference in sample architectures was dictated by the resolution requirements for thickness determination using NR.

### 4.3. FTIR Analysis

To study ionization of PMAA chains within PEM films, PMAA_11_^4min^ and PMAA_11_^24min^ films were deposited onto undoped silicon wafers (University Wafer, Inc., Boston, MA, USA) and FTIR spectra were recorded with a Tensor II spectrophotometer (Bruker Optic GmbH, Ettlingen, Germany). For each sample, 96 scans were recorded between 600 and 4 000 cm^−1^ with 4 cm^−1^ resolution with the standard Bruker OPUS/IR software (version 7.5), using an interferogram of a bare silicon wafer as a background. To obtain and study QPC_12_^4min^ and QPC_12_^24min^ films, one additional layer of the polycation was deposited on top of PMAA_11_^4min^ and PMAA_11_^24min^ multilayers. 

### 4.4. Spectroscopic Ellipsometry

Thicknesses and optical constants in both dry and swollen states of PEMs were characterized by a variable angle spectroscopic ellipsometer (VASE, M-2000 UV-visible-NIR (240−1700 nm) J. A. Woollam Co., Inc., Lincoln, NE, USA) equipped with a temperature-controlled liquid cell. For measurements in the liquid cell, the cell geometry dictated that the angle of incidence be 75°. In all experiments, the temperature was set to 25 °C. To avoid effects of absorption in the ultraviolet and near-infrared light region by the buffer solution, the working wavelength band was set to 370.5–999 nm. Prior to deposition of PEM films, the thickness of the oxide layer on the silicon substrate was measured. Dry measurements were carried out at three angles of incidence: 45°, 55°, and 65°.

To fit the ellipsometric data from dry films, a three-layer model was used, in which the first two layers represented the silicon substrate and its oxide layer, and the third layer represented the PEM film. The polymer layer was treated as a Cauchy material of thickness d, having a wavelength-dependent refractive index *n*(*λ*) = A + B/*λ*^2^ + C/*λ*^3^, where A, B, and C are fitting coefficients, and *λ* is the wavelength. The film extinction coefficient was assumed to be negligible (*k* = 0). Thickness d and the three coefficients A, B, and C were fitted simultaneously. 

For in situ ellipsometry experiments, a silicon wafer with a pre-deposited film of known dry thickness was placed into a liquid cell. The cell was then filled with 0.01 M buffer at pH 6.0 and a thickness measurement was taken. The measurements were finalized after a constant wet thickness was reached and then by removing the sample from the cell, drying with nitrogen flow, and measuring the dry thickness again. To fit the ellipsometric data for the in situ measurements, a four-layer model was used. An additional layer represents the semi-infinite buffer solution and was also treated as a transparent Cauchy medium, with a wavelength-dependent refractive index *n*_buf_(*λ*) = A_buf_ + B_buf_/(*λ*)^2^ + C_buf_/(*λ*)^3^, where A_buf_, B_buf_, and C_buf_ are fitting coefficients, and *λ* is the wavelength. For the buffer solutions, A_buf_, B_buf_, and C_buf_ were determined prior to in situ experiments by measuring *n*_buf_(*λ*) for a bare, clean silicon wafer installed in the liquid cell containing 0.01 M phosphate buffer at pH 6.0. After completion of the in situ measurements, the dry thicknesses of the films were measured again to assure that the thicknesses used in the swelling experiments were consistent with the independently measured dry film thicknesses obtained in the film growth experiments. In all experiments, the coefficients A, B, C were consistent within 5%.

### 4.5. Neutron Reflectometry

Samples prepared for NR studies were assembled using two different designs. In the first design, used for observing the uptake and penetration of *d*QPC chains into hydrogenated matrices, films were assembled using 8 and 24 min immersions (PMAA_11_^8min^ and PMAA_7_^24min^, respectively). Scattering densities (SLDs) for the hydrogenated stacks, upon penetration of a deuterated polyelectrolyte from solution, were held constant for all annealed samples, in fitting varying only the outermost layer’s thickness, interfacial roughnesses, and SLD. In another design, films contained a *d*PMAA block within the middle region of the film, i.e., had QPC_8_/*d*PMAA_9_/QPC_14_^4min^, QPC_8_/*d*PMAA_9_/QPC_14_^8min^, QPC_4_/*d*PMAA_5_/QPC_10_^16min^, or QPC_4_/*d*PMAA_5_/QPC_8_^24min^ architecture.

NR measurements were performed at the Spallation Neutron Source Liquids Reflectometer (SNS-LR) at the Oak Ridge National Laboratory (ORNL). The reflectivity data were collected using a sequence of 3.4-Å-wide continuous wavelength bands (selected from 2.55 Å < *λ* < 16.7 Å) and incident angles (ranging over 0.6° < *θ* < 2.34°). The momentum transfer, *Q* = (4π sin*θ*/*λ*), was varied over a range of 0.008 Å^−1^ < *Q* < 0.193 Å^−1^. Reflectivity curves were assembled by combining seven different wavelength and angle data sets together, maintaining a constant relative instrumental resolution of *δQ*/*Q* = 0.023 by varying the incident-beam apertures. Scattering densities within hydrogenated and deuterated stacks were averaged over the 12 constituent bilayers, with each stack exhibiting its characteristic thickness, scattering-length density, and interlayer roughness. Those characteristic parameters were adjusted until the reflectivity curve was best fitted (minimized *χ*^2^).

## Supplementary Materials

NMR data, the fitting parameters of neutron reflectivity, SLDs, and raw data are available online at www.mdpi.com/2310-2861/4/1/7/s1, Table S1: Model parameters for QPC_8_/*d*PMAA_9_/QPC_14_^4min^ film, Table S2: Model parameters for QPC_8_/*d*PMAA_9_/QPC_14_^8min^ film, Table S3: Model parameters for QPC_4_/*d*PMAA_5_/QPC_10_^16min^ film, Table S4: Model parameters for QPC_4_/*d*PMAA_5_/QPC_8_^24min^ film, Table S5: Model parameters for PMAA_7_^24min^, Table S6: Model parameters for hydrogenated PMAA_7_^24min^ film after 4 min exposure to a 0.2 mg/mL *d*QPC solution, Table S7: Model parameters for hydrogenated PMAA_7_^24min^ film after 8 min exposure to a 0.2 mg/mL *d*QPC solution, Table S8: Model parameters for hydrogenated PMAA_7_^24min^ film after 24 min exposure to a 0.2 mg/mL *d*QPC solution, Table S9: Model parameters for hydrogenated PMAA_11_^8min^ film, Table S10: Model parameters for hydrogenated PMAA_11_^8min^ film after 4 min exposure to a 0.2 mg/mL *d*QPC solution, Table S11: Model parameters for hydrogenated PMAA_11_^8min^ film after 8 min exposure to a 0.2 mg/mL *d*QPC solution, Table S12: Model parameters for hydrogenated PMAA_11_^8min^ film after 24 min exposure to a 0.2 mg/mL *d*QPC solution, Figure S1: Polymerization of DMAEMA (top) and quaternization of hPDMAEMA (bottom), Figure S2: ^1^H-NMR spectra of *h*PDMAEMA before quaternization (**A**) and after complete quaternization and conversion to *h*QPC (**B**) measured in D_2_O at pH 9, Scheme S1: Schematic representation of the layer-by-layer deposition procedure and polymer conformations within films assembled at different deposition time per layer .
