# Supplementary material for: Ionically Paired Layer-by-Layer Hydrogels: Water and Polyelectrolyte Uptake Controlled by Deposition Time
Source: Gels. 2018 Jan 11;4(1):7. doi: 10.3390/gels4010007 (PMC6321383; doi:10.3390/gels4010007)
Supplement: Supplementary file 1 [file gels-04-00007-s001.pdf]

Supporting Information for the Manuscript:

Ionically paired Layer-by-layer Hydrogels: Water and Polyelectrolyte Uptake Controlled by Deposition Time

Victor Selin[1], John F. Ankner[2], and Svetlana A. Sukhishvili[1]*

[1] *Department of Materials Science & Engineering, Texas A&M University, College Station, Texas 77843, USA*

[2]*Spallation Neutron Source, Oak Ridge National Laboratory, Oak Ridge, Tennessee 37831, USA*

Email: svetlana@tamu.edu

Table S1. Model parameters for $QPC_8/dPMAA_9/QPC_{14}^{4min}$ film.

**Layer**	***Nb*** **(Å^{-2})**	***d*** **(Å)**	σ_{int} **(Å)**
H-stack	6.430E-07	457.0	47.6
D-stack	2.330E-06	143.4	66.8
H-stack	6.430E-07	478.3	57.2
BPEI	2.500E-07	29.8	29.8
SiO2	3.200E-06	18.0	5.0
Si	2.070E-06	100.0	5.0

Table S2. Model parameters for $QPC_8/dPMAA_9/QPC_{14}^{8min}$ film.

**Layer**	***Nb*** **(Å^{-2})**	***d*** **(Å)**	σ_{int} **(Å)**
H-stack	6.430E-07	477.4	139.0
D-stack	2.405E-06	260.0	166.0
H-stack	6.443E-07	404.0	118.8
BPEI	2.500E-07	75.0	75.0
SiO2	3.200E-06	18.0	5.0
Si	2.070E-06	100.0	5.0

Table S3. Model parameters for $QPC_4/dPMAA_5/QPC_{10}^{16min}$ film.

Layer	*Nb* (Å^{-2})	*d* (Å)	σ_{int} (Å)
H-stack	5.33E-07	652.6	165.6
D-stack	2.30E-06	270.1	178.0
H-stack	6.39E-07	290.5	120.0
BPEI	3.12E-07	15.9	5.0
SiO2	3.40E-06	26.1	16.3
Si	2.07E-06	100.0	19.0

Table S4. Model parameters for $QPC_4/dPMAA_5/QPC_8^{24min}$ film.

Layer	*Nb* (Å^{-2})	*d* (Å)	σ_{int} (Å)
H-stack	7.550E-07	314.0	270.6
D-stack	2.287E-06	581.7	176.2
H-stack	1.550E-06	115.5	109.9
BPEI	3.500E-07	5.0	5.0
SiO2	3.200E-06	25.0	18.0
Si	2.070E-06	100.0	25.0

Table S5. Model parameters for $PMAA_7^{24min}$.

Layer	*Nb* (Å^{-2})	*d* (Å)	σ_{int} (Å)
H-stack	6.430E-07	540.0	65.0
BPEI	3.790E-07	28.0	5.0
SiO2	3.200E-06	19.0	10.0
Si	2.070E-06	100.0	19.0

Table S6. Model parameters for hydrogenated $PMAA_7^{24min}$ film after 4 min exposure to a 0.2 mg/ml *d*QPC solution.

Layer	*Nb* (Å^{-2})	*d* (Å)	σ_{int} (Å)
D-stack	1.980E-06	500.5	80.0
H-stack	6.430E-07	95.0	95.0
BPEI	3.790E-07	75.0	30.6
SiO2	3.200E-06	18.7	5.0
Si	2.070E-06	100.0	18.7

Table S7. Model parameters for hydrogenated $PMAA_7^{24min}$ film after 8 min exposure to a 0.2 mg/ml *d*QPC solution.

Layer	***Nb*** **(Å^{-2})**	***d*** **(Å)**	σ_{int} **(Å)**
D-stack	2.050E-06	506.4	79.3
H-stack	6.430E-07	88.7	88.7
BPEI	3.790E-07	75.0	30.6
SiO2	3.200E-06	18.7	5.0
Si	2.070E-06	100.0	18.7

Table S8. Model parameters for hydrogenated $PMAA_7^{24min}$ film after 24 min exposure to a 0.2 mg/ml *d*QPC solution.

Layer	***Nb*** **(Å^{-2})**	***d*** **(Å)**	σ_{int} **(Å)**
D-stack	1.953E-06	521.6	80.7
H-stack	6.430E-07	92.0	92.0
BPEI	3.790E-07	75.0	30.0
SiO2	3.200E-06	18.7	5.0
Si	2.070E-06	100.0	18.7

Table S9. Model parameters for hydrogenated $PMAA_{11}^{8min}$ film.

Layer	***Nb*** **(Å^{-2})**	***d*** **(Å)**	σ_{int} **(Å)**
H-Block	6.430E-07	1002.0	120.0
BPEI	3.790E-07	28.0	5.0
SiO2	3.200E-06	19.0	10.0
Si	2.070E-06	100.0	19.0

Table S10. Model parameters for hydrogenated $PMAA_{11}^{8min}$ film after 4 min exposure to a 0.2 mg/ml *d*QPC solution.

**Layer**	***Nb*** **($Å^{-2}$)**	***d*** **(Å)**	**σ_{int} (Å)**
D-block	7.000E-07	430.5	85.0
H-block	6.430E-07	556.6	180.0
BPEI	4.500E-07	73.5	62.5
SiO2	3.400E-06	16.0	5.0
Si	2.070E-06	100.0	5.0

Table S11. Model parameters for hydrogenated $PMAA_{11}^{8min}$ film after 8 min exposure to a 0.2 mg/ml *d*QPC solution.

**Layer**	***Nb*** **($Å^{-2}$)**	***d*** **(Å)**	**σ_{int} (Å)**
D-block	8.000E-07	589.7	100.8
H-block	6.430E-07	419.7	177.7
BPEI	5.000E-07	67.9	67.0
SiO2	3.400E-06	16.0	5.0
Si	2.070E-06	100.0	5.0

Table S12. Model parameters for hydrogenated $PMAA_{11}^{8min}$ film after 24 min exposure to a 0.2 mg/ml *d*QPC solution.

**Layer**	***Nb*** **($Å^{-2}$)**	***d*** **(Å)**	**σ_{int} (Å)**
D-block	1.080E-06	660.0	155.0
H-block	6.430E-07	352.0	325.9
BPEI	2.700E-07	75.0	62.5
SiO2	3.400E-06	16.0	5.0
Si	2.070E-06	100.0	5.0

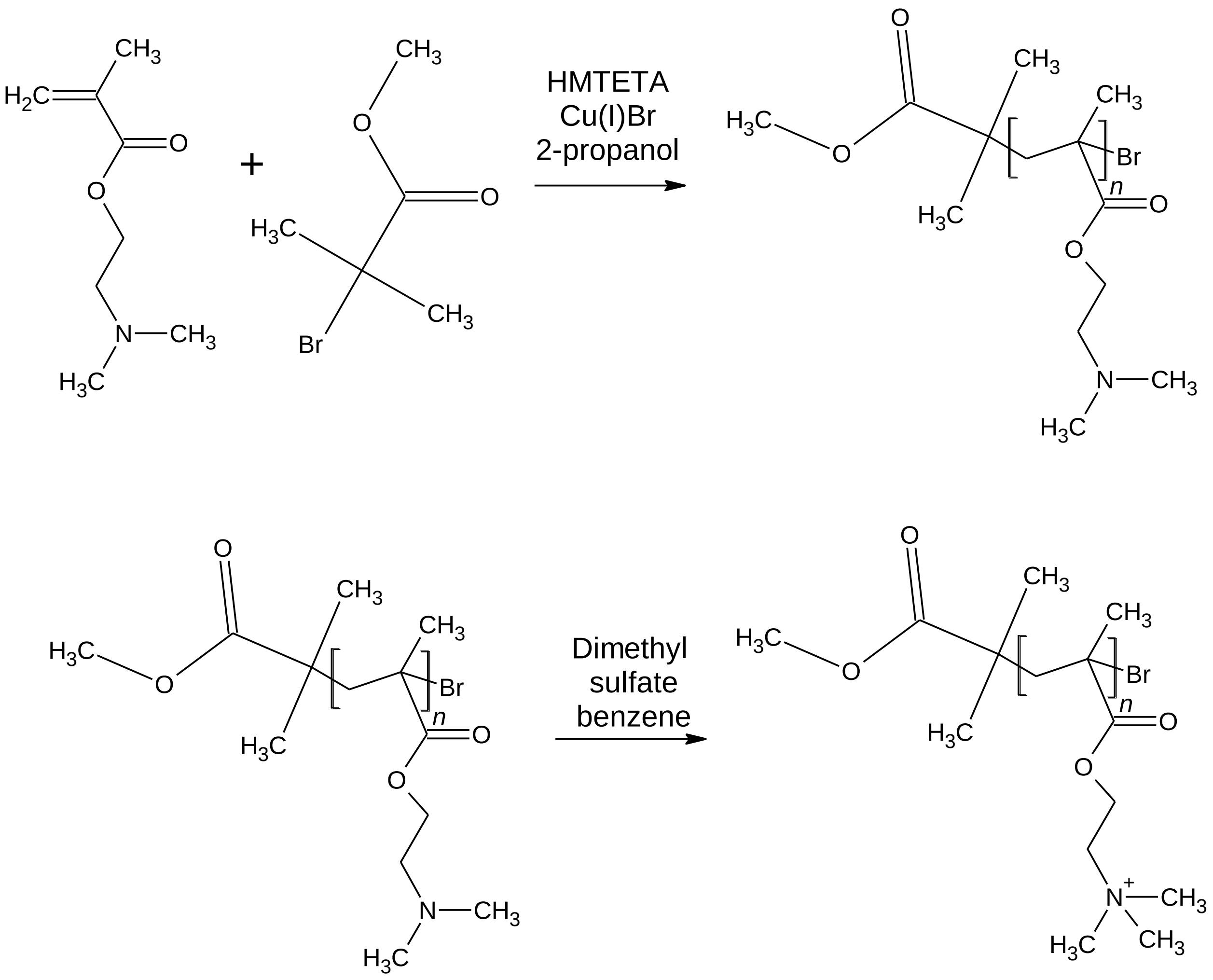

Fig S1. Polymerization of DMAEMA (top) and quaternization of *h*PDMAEMA (bottom).

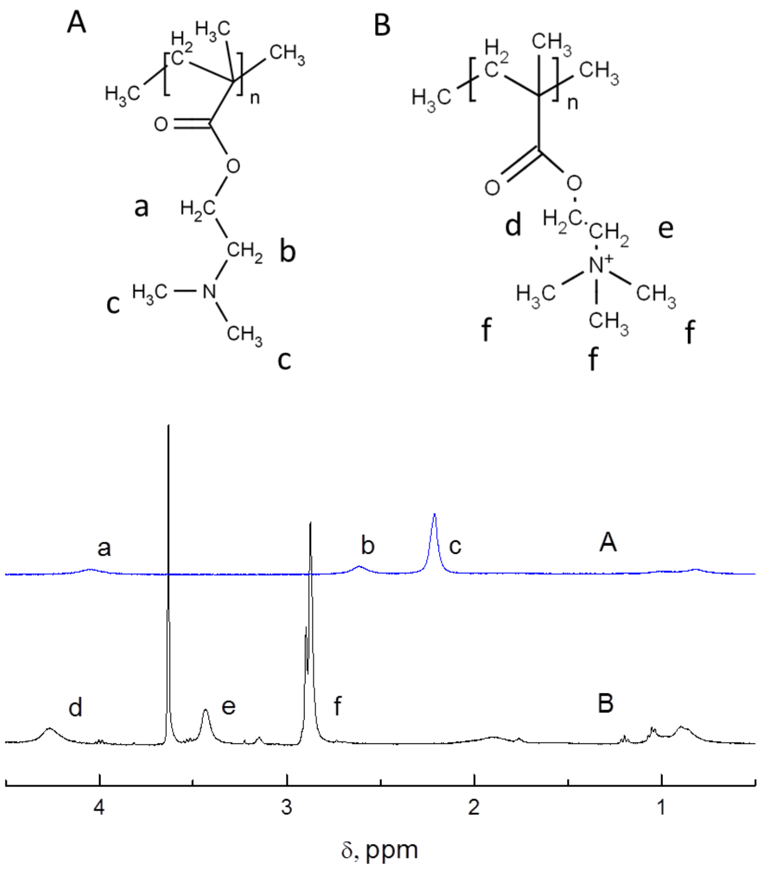

Fig. S2. ^{1}H-NMR spectra of *h*PDMAEMA before quaternization (A) and after complete quaternization and conversion to *h*QPC (B) measured in D_2O at pH 9.

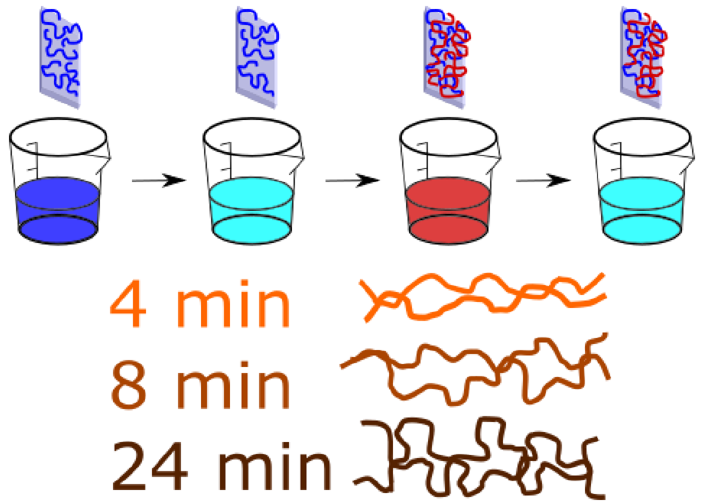

Scheme S1. Schematic representation of the layer-by-layer deposition procedure and polymer conformations within films assembled at different deposition time per layer.
